# Megarbane syndrome

**DOI:** 10.4103/0971-6866.42325

**Published:** 2008

**Authors:** Caglayan Ahmet Okay, Dundar Munis

**Affiliations:** Erciyes University Medical Faculty Department of Medical Genetics, Kayseri, Turkey

**Figure 1:**
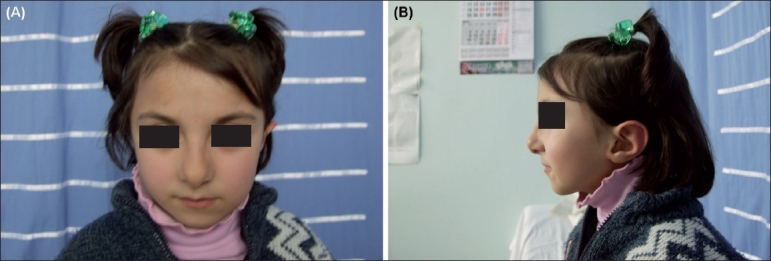
(A) Facial appearance of case at age 6 years. Note microcephaly. (B) Profile of case. Note micrognathia, low set ears

Sir,

Megarbane *et al.* reported two male cousins from a consanguineous family.[1] In their cases syndrome consisting of minor facial anomalies, microcephaly, colobomatous microphthalmia, psychomotor retardation, short stature, and skeletal malformations. We describe a new case who has megarbane diagnosis according to her clinical findings. Present case [Figure 1] is the only child of healthy nonconsanguineous divorced parents. Gestation was unremarkable. She was delivered by normal spontaneous vaginal way with siyanose at 38 weeks. Birth weight was 3800 g. Other measurements were unknown. When she was came to us she at the age of 6. She was 15 kg weight, 101 cm length, and an occipitofrontal circumference (OFC) of 47 cm (all below the 3rd centile). She had microcephaly, coloboma of the iris in left eye, strabismus, and mental retardation. Her blood glucose levels, urine analysis, aminoacid studies of plasma and urine, and liver and thyroid function studies, and other routin tests were unremarkable. Magnetic resonance (MR) imaging of the brain showed coloboma of the optic disc. Echocardiogram (ECHO) was normal. Evoked response audiometry (ERA) test revealed that left ear had a total hearing loss with unknown etiology and right ear had normal hearing. Eye consultation resulted, esotrophia in left eye, bilaterally optical disc coloboma, and coloboma of the iris in left eye. Denver developmental test showed mild mental retardation (IQ;52). Chromosomes (high-resolution G- and R-banding) were normal (46,XX). Her parents physical findings were normal. Some of these findings are also seen in, recessive Waardenburg microphthalmia syndrome, CHARGE syndrome, Lenz microphthalmia, and Megarbane syndrome [Table 1].

**Table 1:** We compared some features of the present case with possible syndromes

Features	Megarbane syndrome	Recessive Waardenburg microphthalmia syndrome	CHARGE syndrome	Lenz microphthalmia syndrome	Present case
Craniofacial dysmorphism	+	+	+		+
Hearing impairment		+	+		+
Anophthalmia or microphthalmia	+	+		+	+
Coloboma	+		+	+	+
Choanal atresia			+		
Microcephaly	+			+	+
Mental retardation	+	+		+	+
Retarded growth and development	+	+	+	+	+
Heart defect	+		+	+	
Pigmentation defects		+			
Skeletal anomalies	+		+	+	
Urogenital anomalies			+	+	

Waardenburg syndrome (WS) is a rare autosomal-dominant condition characterized by sensory/neural hearing loss, pigmentary abnormalities of the skin, hair, and eyes, and craniofacial anomalies.[2] Our patient's clinical findings are similar with the WSII, but she has not pigmentary abnormalities of the skin, hair, craniofacial anomalies, hands, and feet malformations.

CHARGE syndrome was first described in 1979 by Hall *et al.*[3] in 17 children with multiple congenital abnormalities detected on the basis of choanal atresia. In 1981, Pagon *et al.*[4] proposed the acronym CHARGE (coloboma, heart defect, choanal atresia, retarded growth and development, genital hypoplasia, ear abnormalities, and/or hearing loss). Diagnosis is based on major (coloboma, choanal atresia, characteristic ear abnormalities, cranial nerve dysfunction, and temporal bone abnormality) and minor (genital hypoplasia, developmental delay, cardiac abnormality, growth retardation, orofacial cleft, tracheoesophageal fistula, and distinctive face) criteria. CHARGE syndrome is defined as a combination of the five major criteria or four major and three of the seven minor criteria, according to two landmark studies.[5,6] Our patient does not fulfill these criteria.

Another rare condition that could be considered in the differential diagnosis is Al Frayh-Anophthalmia, microcephaly, hypogonadism, MR syndrome. Al Frayh and Haque[7] described a mentally retarded boy with anophthalmia/microphthalmia, hypotonia, coloboma of the iris, microcephaly, hypogonadism,failure to thrive, cardiac malformation, and short stature, but our patient hasn't hypogonadism, hypotonia, cardiac malformation and anophtalmia.

Lenz syndrome is a rare X-linked recessive condition first reported by Lenz.[8] All affected individuals have mental retardation, mild-to-severe microphthalmos, with colobomas in about 75% of cases. Our patients also have coloboma, microcephaly, mental retardation, and short stature. Carrier females in Lenz microphthalmia syndrome may have short stature, microcephaly, and mental retardation.[9 10] The absence of these manifestations in the mothers of our patients could be explained by the Lyon hypothesis. Nevertheless, the lack of some major components of Lenz syndrome[11] such as dental, digital, and urogenital anomalies, in addition to the severe mental retardation, distinct facial appearance, make a diagnosis of Lenz syndrome unlikely.

Although our patient has not skeletal malformations and inheritance of this case is uncertain, other many features thinking Megarbane syndrome. We described this case as a second Megarbane syndrome paper to the literature.
